# Technikgestützte zahnmedizinische Hausbesuche durch nicht-ärztliches Fachpersonal zur Minderung des Ansteckungsrisikos

**DOI:** 10.1365/s40702-021-00733-z

**Published:** 2021-05-26

**Authors:** Sarah-Sabrina Kortekamp, Ingmar Ickerott, Frank Teuteberg

**Affiliations:** 1 Institut für Management und Kultur, Hochschule Osnabrück, Lingen, Niedersachsen Deutschland; 2 Fachgebiet für Unternehmensrechnung und Wirtschaftsinformatik, Universität Osnabrück, Osnabrück, Niedersachsen Deutschland

**Keywords:** Designprinzipien, Mixed Reality, Virtual Reality, Head-Mounted Devices, Zahnmedizinische Hausbesuche, Design principles, Mixed reality, Virtual reality, Head-mounted devices, Dental home visits

## Abstract

Ziel des Beitrags ist die Identifikation von Problemen, Meta-Anforderungen und Designprinzipien für den Einsatz von Mixed und Virtual Reality Brillen zur Unterstützung nicht-ärztlichen Fachpersonals bei zahnmedizinischen Hausbesuchen. Im Rahmen von zwei Gruppendiskussionen und einem Experteninterview wurden zunächst mögliche Einsatzszenarien identifiziert. Anschließend wurde eine systematische Literaturrecherche in den Datenbanken CINAHL, Business Source Premier und MEDLINE durchgeführt. In der gefundenen Literatur konnten 14 Probleme bei der Anwendung von Mixed und Virtual Reality Brillen identifiziert werden. Darauf basierend wurden 14 Meta-Anforderungen abgeleitet und in fünf Designprinzipien zusammengefasst. Abschließend wurden die Ergebnisse mit den Spezifikationen der Microsoft HoloLens 2 abgeglichen, um eine Eignung für die Unterstützung der geplanten Hausbesuche festzustellen. Zudem wurde ein Umsetzungskonzept skizziert. Die Ergebnisse dienen als wichtige Empfehlungen für die praxisnahe Umsetzung zukünftiger Konzepte bezüglich der Anwendung von Mixed und Virtual Reality Brillen im (zahn-)medizinischen Kontext. Die Literaturrecherche zeigt eine Forschungslücke im Bereich zahnmedizinischer Hausbesuche auf. Die Ergebnisse dieses Beitrags schaffen daher eine solide Basis für die zukünftige Forschung.

## Abstract (en)

The aim of this paper is to identify problems, meta-requirements and design principles for the use of mixed and virtual reality glasses to support nondoctoral professionals during dental home visits. In the course of two group discussions and one expert interview, possible application scenarios were first identified. Subsequently, a systematic literature search was conducted in the databases CINAHL, Business Source Premier and MEDLINE. The literature revealed 14 problems in the application of mixed or virtual reality glasses. Based on this, 14 meta-requirements were derived and summarised in five design principles. Finally, the results were compared with the specifications of the Microsoft HoloLens 2 to determine suitability. An implementation concept was also outlined. The results serve as important recommendations for the practical implementation of future concepts regarding the application of mixed and virtual reality glasses in a (dental) medical context. The literature review reveals a research gap in the field of dental home visits. Therefore, the results of this paper provide a solid basis for future research.

## Einleitung

Aufgrund der Corona-Pandemie wurden in vielen Ländern Maßnahmen getroffen, die eine Ausbreitung von Covid-19 möglichst eindämmen sollen. Bestimmte Personengruppen, wie ältere Menschen mit chronischen Erkrankungen, sind durch ein erhöhtes Infektionsrisiko und möglicherweise schwere Krankheitsverläufe besonders betroffen (Jordan et al. 2020; Zhou et al. 2020).

Der Besuch beim Zahnarzt stellt für diese Personengruppen durch den engen Kontakt zu anderen Personen trotz sorgfältiger Hygienemaßnahmen ein erhöhtes Ansteckungsrisiko dar. Ein Auslassen der Kontrolltermine oder das Ignorieren von Beschwerden kann allerdings ebenso gravierende Folgen haben. So können Infektionen im Mundraum durch eine Verschleppung von Keimen beispielsweise eine Aspirationspneumonie auslösen (Baumgartner et al. 2015).

Hausbesuche könnten hier eine mögliche Alternative darstellen, die eine zahnmedizinische Versorgung ermöglicht und dabei das Ansteckungsrisiko vulnerabler Personen möglichst minimiert. Die Durchführung von zusätzlichen Hausbesuchen stellt aber auch gleichzeitig eine höhere Belastung des zahnärztlichen Personals dar.

Im hausärztlichen Bereich existieren bereits seit Längerem Fortbildungen für nicht-ärztliche Praxisassistenzen. Dies ermöglicht die Delegation von ansonsten ärztlichen Tätigkeiten an nicht-ärztliches medizinisches Personal. Nichtärztliches Fachpersonal kann so verschiedene Maßnahmen im Rahmen von Hausbesuchen selbstständig durchführen (Gisbert Miralles et al. 2020). Dieses Angebot besteht allerdings noch nicht für zahnmedizinische Praxen (BARMER Internetredaktion 2020).

Auch in der Forschung ist dieses Thema kaum repräsentiert. Eine explorative Literaturrecherche mithilfe von Google Scholar ergab zwar viele Ergebnisse zum Einsatz von Mixed Reality (MR) Brillen zur Ausbildung oder Unterstützung während Operationen im zahnmedizinischen Bereich. Wissenschaftliche Beiträge zu Hausbesuchen durch nicht-ärztliches Fachpersonal im Allgemeinen oder in Verbindung mit MR Brillen konnten nicht gefunden werden.

Das Ziel dieser Forschung ist daher die Erprobung von Hausbesuchen durch nicht-ärztliches zahnmedizinisches Personal. Dabei soll beispielhaft die Microsoft HoloLens 2 unterstützend eingesetzt werden. Die Forschungsfragen dieses Beitrags lauten:

- Welche Behandlungen können im Rahmen eines zahnmedizinischen Hausbesuchs mit nicht-ärztlichem Fachpersonal und unterstützt durch MR Brillen durchgeführt werden?
- Welche Anforderungen stellt dieses Nutzungsszenario an Hardware und Software?
- Inwiefern wird die Microsoft HoloLens 2 diesen Anforderungen gerecht?

Im nächsten Abschnitt wird das methodische Vorgehen beschrieben, bevor die Ergebnisse im dritten Abschnitt in Form von Einsatzszenarien, Designprinzipien und einem Umsetzungskonzept dargestellt werden. Im letzten Abschnitt werden die Ergebnisse, die Limitationen des Beitrags sowie die praktische und theoretische Signifikanz diskutiert.

## Methode

Im Rahmen des Design Science Ansatzes (Gregor and Hevner 2013) wird ein iteratives Vorgehen anvisiert. Entsprechend werden bestehende Ergebnisse fortlaufend überprüft und im Falle neu gewonnener Erkenntnisse überarbeitet.

### Gruppendiskussion und Experteninterview

Zur Beantwortung der ersten Frage wurden zwei interdisziplinäre, formal geleitete Gruppendiskussionen durchgeführt (Flick 2009). Unter den fünf und acht Teilnehmenden befanden sich ein Zahnarzt und Personen aus den Bereichen der Informations- und Kommunikationstechnik, der Wirtschaftsinformatik sowie der Ergonomie. Die erste Gruppendiskussion diente dem Finden möglicher Einsatzszenarien für nicht-ärztliche zahnmedizinische Hausbesuche mit Unterstützung durch die Microsoft HoloLens 2. Die darauffolgende Gruppendiskussion sowie ein halbstrukturiertes Experteninterview mit einem praktizierenden Zahnarzt dienten der Konkretisierung der sondierten Szenarien.

### Systematische Literaturrecherche

Um die zweite Frage zu beantworten, wurde eine systematische Literaturrecherche durchgeführt (vom Brocke et al. 2015). Dafür wurden CINAHL und Business Source Premier als relevante Datenbanken für die Wirtschaftsinformatik ausgewählt. Aufgrund des medizinischen Themas wurde MEDLINE in die Recherche eingeschlossen. Eine Fokussierung auf MR in einem zahnmedizinischen Kontext war infolge der geringen relevanten Treffer nicht möglich. Aufgrund der thematischen Verwandtschaft wurde daher auch Literatur inkludiert, die sich mit Virtual Reality (VR) in einem generellen medizinischen Setting beschäftigt.

Für die Suche wurden die Begriffe „Augmented Reality“, „Mixed Reality“ und „Virtual Reality“ mit den Begriffen „dental*“, „dentist*“, „medicine“, „care“ und „telehealth“ kombiniert. Die gefundenen Beiträge wurden systematisch auf die Nennung von Problemen bei der Nutzung von Mixed oder Virtual Reality Brillen untersucht. Inklusionskriterien waren die Nutzung einer solchen Brille durch medizinisches Fachpersonal. Aus- und Weiterbildungsszenarien wurden aufgrund zu unterschiedlicher Parameter ausgeschlossen. Insgesamt konnten zehn relevante Beiträge identifiziert werden.

Zur Beantwortung der dritten Frage wurden die identifizierten Meta-Anforderungen und Designprinzipien mit den Eigenschaften der Microsoft HoloLens 2 verglichen (s. Abschn. 3.3).

## Ergebnisse

### Einsatzszenarien

Es konnten insgesamt fünf Einsatzszenarien für die Anwendung von MR Brillen bestimmt werden, die in Tab. 1, Spalte 1, dargestellt sind. In der zweiten Spalte werden die Mindestanforderungen an die Qualifikation beschrieben und in Spalte 3 das Equipment, das zusätzlich zur MR Brille erforderlich ist. Im Folgenden werden die Szenarien dezidiert beschrieben.

**Tab. 1:** Mögliche Einsatzszenarien einer Mixed Reality Brille während eines zahnmedizinischen Hausbesuchs

Nr	Einsatzszenario	Nutzende	Equipment
1	Video-Dokumentation des Mundraums	Med./pfleg. Fachpersonal	Zahnmedizinisches Grundbesteck
2	Regelmäßige Kontrolle; Erfassung eines zahnmedizinischen Problems	Med./pfleg. Fachpersonal mit Weiterbildung, zahnmedizinisches Fachpersonal	Zahnmedizinisches Grundbesteck
3	Behebung von Prothesendruckstellen	Erfahrene zahnmedizinisches Fachpersonal	Zahnmedizinisches Grundbesteck, Fräse
4	Reparatur eines abgebrochenen Zahns (Prothese)	Zahntechniker/in	Individuelles zahntechnisches Equipment
5	Prophylaxe	Zahnmedizinische Prophylaxehelfer/in	Mobile Dentaleinheit

Das erste Szenario befasst sich mit der Video-Dokumentation des Mundraums durch med./pfleg. Fachpersonal mithilfe einer MR Brille. Ziel ist es, diese Untersuchung vornehmen zu können, wenn med./pfleg. Fachpersonal aufgrund eines anderen Problems bereits bei der zu behandelnden Person zu Hause ist. Das zuständige zahnärztliche Fachpersonal kann die Videoaufnahme im Anschluss in der Praxis begutachten und feststellen, ob zahnmedizinische Probleme vorliegen.

Med./pfleg. Fachpersonal mit Weiterbildung könnte, in einem gut eingespielten interdisziplinären Team zudem regelmäßige Kontrollen der Zähne selbstständig durchführen oder auf Anfrage ein bestimmtes zahnmedizinisches Problem erfassen (Szenario 2). Die Durchführung durch med./pfleg. Fachpersonal hat auch hier wieder den Vorteil, dass die Untersuchung mit anderen Tätigkeiten kombiniert werden kann. Die MR Brille dient dann der Dokumentation und bei Bedarf auch der Absprache mit dem zuständigen zahnärztlichen Fachpersonal.

Im Rahmen des dritten Szenarios nimmt erfahrenes zahnmedizinisches Fachpersonal Anpassungen am Sitz der Prothese der zu behandelnden Person vor, um so Druckstellen zu beheben. Zusätzlich zur MR Brille und einem zahnmedizinischen Grundbesteck wird dazu eine Fräse benötigt.

Szenario vier beschreibt die Reparatur eines abgebrochenen Zahns aus einer Prothese. Diese Maßnahme kann von einer/einem Zahntechniker/in durchgeführt werden, wobei individuelles zahntechnisches Equipment vonnöten ist.

Das letzte Szenario ist die Durchführung der Prophylaxe durch eine/n zahnmed. Prophylaxehelfer/in. Im Gegensatz zu den anderen Szenarien ist für diese Tätigkeit eine mobile Dentaleinheit erforderlich. Die MR Brille dient hier, zusätzlich zu Dokumentation und Rücksprache mit dem zuständigen zahnärztlichen Fachpersonal, auch der Kontrolle des Ergebnisses.

### Probleme, Meta-Anforderungen und Designprinzipien

Aus den, in der Literatur identifizierten, Problemen (*P*) wurden die Meta-Anforderungen (*MA*) entwickelt. Die Designprinzipien (*DP*) basieren auf diesen Erkenntnissen und fassen die Meta-Anforderungen praxisnah zusammen. Die Designprinzipien dienen der Eignungsprüfung der Microsoft HoloLens 2. Die genannten Probleme beziehen sich zum einen auf die Eigenschaften der MR Brillen selbst. Zum anderen auch auf die Interaktion mit der Technik sowie die Einbindung in die bestehende Infrastruktur.

#### Techniklimitationen

Bezüglich der Hardware wurde mehrfach die kurze Batterielaufzeit (*P1*) (Sparwasser et al. 2018; Martin et al. 2020; Galati et al. 2020) bemängelt. Dies ist besonders beim Einsatz in der Chirurgie bemerkbar, da die derzeitigen Modelle mit einer Batterielaufzeit von zwei bis drei Stunden nur während kürzerer Eingriffe die gesamte Zeit über unterstützen können. Hinzu kommt die begrenzte Speicherkapazität (Talaat et al. 2019; Galati et al. 2020) und Rechenkapazität der mobilen Prozessoren (*P2*) (Sparwasser et al. 2018; Galati et al. 2020). Die Spezifikationen des Geräts sollten daher auf die geplante Nutzung abgestimmt werden. Bei Bedarf sollte das Gerät während der Nutzung komfortabel geladen werden können oder einen austauschbaren Akku besitzen (*MA1*). Bei stationären Einsatzszenarien und einem geeigneten Netzwerk sollten ressourcenintensive Prozesse in die Cloud ausgelagert werden können (*MA2*). In stationären Szenarien wären prinzipiell auch MR Brillen einsetzbar, die in Verbindung mit einem externen Computer genutzt werden müssen. Hier treffen die oben genannten Probleme nicht zu. Allerdings erwies sich die, für die Betreibung, notwendige Infrastruktur von Computer, Stromanschluss und (Verlängerungs-)kabeln im Einsatz als unpraktisch (*P3*) (Sparwasser et al. 2018; Moosburner et al. 2019).

##### *DP 1*

Die Hardwarespezifikationen sollten an die individuelle Umgebung des jeweils aktuellen medizinischen Nutzungskontexts (wie z. B. bestehende Infrastruktur und Einsatzszenario) angepasst sein.

#### Ergonomie

Ebenfalls wurde die ungünstige Gewichtsverteilung der MR Brillen bemängelt (*P4*). So wirkte sich das frontlastige Gewicht der Microsoft HoloLens 1 bei längerem Einsatz negativ auf die Körperhaltung der Nutzenden aus (Galati et al. 2020). Auch die Aufnahme von Fotos mit der integrierten Kamera führte aufgrund der ungünstigen Positionierung zu Problemen mit der Körperhaltung (*P5*) (Galati et al. 2020). Daraus ergibt sich, dass die Konstruktion der Hardware an die Nutzung und auch die, für die Tätigkeit spezifische Körperhaltung, angepasst sein sollte (*MA3*).

##### *DP 2*

Die ergonomischen Eigenschaften der Hardware sollten für die, während der medizinischen Tätigkeiten notwendige Körperhaltung geeignet sein.

#### Nutzerzentrierte Entwicklung

Bezüglich der Funktionalität der MR Brille wurde berichtet, dass beim Einsatz für Tele-Visiten bei zu behandelnden Personen die Kommunikation zwischen Tele-Arzt bzw. Tele-Ärztin und der zu behandelnden Person schwierig ist, da letztere das per Videoanruf zugeschaltete Personal unter Umständen nicht hören kann (*P6*) (Martin et al. 2020). Daher ist es notwendig, die Möglichkeit zu schaffen, die interne Tonausgabe auf die externe oder eine geteilte Tonausgabe umstellen zu können (*MA4*).

MR Brillen führen in der Regel eine Kalibrierung der Optik auf den aktuellen Nutzenden durch. Ziel ist u. a. die Verbesserung der optischen Darstellung virtueller Inhalte. Der Vorgang wurde jedoch als umständlich und teils auch ineffektiv beschrieben (*P7*) (Moosburner et al. 2019). Daher sollte überlegt werden, ob der Aufwand einer Kalibrierung tatsächlich den Nutzen rechtfertigt. Insbesondere im gewerblichen Umfeld, in dem sich mehrere Nutzende eine Brille teilen, kann sich die, für die Kalibrierung benötigte Zeit, schnell aufsummieren. Sofern eine Kalibrierung notwendig ist, sollte diese also einfach, schnell und zuverlässig durchführbar sein. Das Ergebnis sollte in einem Nutzerprofil gespeichert werden, sodass jeder Nutzende die Kalibrierung nur einmal bei Erstnutzung des Geräts durchführen muss (*MA5*).

Gerade im medizinischen Bereich ist die freihändige Bedienung der MR Brillen ein großer Vorteil. So kann das Gerät auch während Arbeiten genutzt werden, in denen Nutzende steril bleiben müssen. Allerdings wurde das fehlerhafte Erkennen von Sprach- und Gesteneingaben als bestehendes Problem berichtet, das bei Nutzenden zu Frustration führte (*P8*) (Moosburner et al. 2019; Galati et al. 2020). Zur Vorbeugung sollten die unterstützten Steuerungsmöglichkeiten einwandfrei funktionieren (*MA6*).

##### *DP 3*

Das Feedback medizinischen Fachpersonals sollte bereits früh in die funktionale Softwareentwicklung integriert werden, um damit eine nutzerzentrierte Umsetzung geeigneter Funktionen und Abläufe zu ermöglichen und sicherzustellen, dass das Hardware‑/Softwaresystem funktioniert, wie von den Nutzenden erwartet.

#### Orientierung im virtuellen Raum

Hauptaufgabe der MR Brille ist die Darstellung von virtuellen Inhalten, wie Text, Video oder 3D Objekten. Diese überlagern die Realität mit zusätzlichen Informationen, um den Nutzenden bei ihren Aufgaben zu unterstützen. Der Bereich, in dem aktuelle MR Brillen virtuelle Inhalte darstellen können ist allerdings merklich kleiner als das Sichtfeld eines Menschen. Dies führt teilweise zu einer ungünstigen Positionierung virtueller Objekte im Sichtfeld (Pellegrino et al. 2019) und zu Orientierungsproblemen in der virtuellen Umgebung (*P9*) (Moosburner et al. 2019; Galati et al. 2020). Um diesem Problem vorzubeugen, müssen die virtuellen Inhalte frei positionierbar sein (*MA7)*. Des Weiteren sollte auf virtuelle Inhalte, die sich gerade nicht im Sichtfeld befinden, eindeutig hingewiesen werden (*MA8*).

Kanevsky et al. (2019) beschreiben, dass die Nutzung von MR Brillen während eines chirurgischen Eingriffs zu einer schlechteren Reaktionszeit auf unvorhersehbare Ereignisse führt (*P10*). Entsprechend sollten die virtuellen Elemente schnell und problemlos aus- und eingeblendet werden können (*MA9*).

##### *DP 4*

Um eine intuitive Orientierung im virtuellen Raum zu ermöglichen und die Lokalisierung von Interfaceelementen zu erleichtern, sollte das virtuelle Sichtfeld möglichst Deckungsgleich mit dem menschlichen sein. Die Positionierung virtueller Objekte sollten Nutzende jederzeit anpassen können.

#### Infrastruktur

Des Weiteren wurden auch Probleme genannt, die sich mit der Vorbereitung und Wartung der MR Brillen beschäftigen. Ein wesentlicher Aspekt ist hier der hohe zeitliche Aufwand, der in die Aufbereitung der Bilddaten für die 3D Anwendung investiert werden muss (*P11*) (Talaat et al. 2019; Pellegrino et al. 2019; Goo et al. 2020). Dies ist u. a. auch der Tatsache geschuldet, dass hierfür mehrere verschiedene Softwareapplikationen notwendig sind. Für einen breiten Einsatz von MR Brillen ist hier die Entwicklung spezifisch zugeschnittener Software sinnvoll, um den Einstieg zu vereinfachen (*MA10*). Ein weiteres Problem, das vorwiegend telemedizinische Anwendungen betrifft, ist die Netzwerkverbindung. Bezüglich der Netzwerkgeschwindigkeit und -kapazität traten vermehrt Stabilitäts- und Konnektivitätsprobleme auf (*P12*) (Martin et al. 2020). Abgesehen vom Ausbau der notwendigen Infrastruktur (*MA11*) sollte auf eine hohe Resilienz gegenüber Netzwerkschwankungen geachtet werden (*MA12*). Die MR Brille sollte nach einem Verbindungsabbruch einen schnellen Verbindungsneuaufbau forcieren. Zudem sollten alle Daten auf dem Gerät automatisch und in kurzen Intervallen zwischengespeichert werden, um Datenverlust zu vermeiden.

Ein weiteres Problem ist die Vulnerabilität personenbezogener Daten auf einem mobilen Gerät (*P13*) (Carenzo et al. 2015; Sparwasser et al. 2018; Kanevsky et al. 2019) und auch auf das Schadenpotential von Malware in Verbindung mit virtuellen Überlagerungen der realen Welt wurde hingewiesen (*P14*) (Kanevsky et al. 2019; Persky und Lewis 2019). Entsprechend wichtig ist die Einhaltung aktueller Standards zu Datenschutz und -sicherheit (*MA13*). Aufgrund der relativ neuen Technik in Verbindung mit neuen Anwendungsgebieten ist es außerdem essenziell, die neuen Anforderungen zu begutachten und auf die geänderten Bedürfnisse abgestimmte Standards zu entwickeln (*MA14*).

##### *DP 5*

Für die Schaffung eines Umfelds, in dem das Hardware‑/Softwaresystem effizient, effektiv und zufriedenstellend nutzbar ist, sollte zum einen die Software eine hohe Resilienz gegenüber Netzwerkproblemen aufweisen. Zum anderen sollten entsprechende Infrastrukturen organisationsintern, als auch in der Fläche geschaffen werden.

Abschließend muss noch ein Problem ohne derzeit bestehende Lösung genannt werden. Der „Parallax Error“ (Galati et al. 2020) bezieht sich auf einen Anzeigefehler von virtuellen Inhalten. Von verschiedenen Winkeln aus betrachtet, können technisch bedingt virtuelle Punkte um mehrere Zentimeter verschoben dargestellt werden (Moosburner et al. 2019; Goo et al. 2020; Galati et al. 2020).

### Mögliche Umsetzung mit der Microsoft HoloLens 2

Die Einsatzszenarien wurden mit Blick auf die funktionellen Möglichkeiten der Microsoft HoloLens 2 formuliert. Im Folgenden sollen die identifizierten Meta-Anforderungen und Designprinzipien auf diese MR Brille angewandt werden, um so eine Eignung für die oben beschriebenen Einsatzszenarien sicherzustellen.

Das Sichtfeld hat sich im Vergleich zur ersten Generation deutlich vergrößert; entspricht jedoch noch nicht dem des menschlichen Sichtfeldes. Hologramme sind allerdings frei positionierbar und das Visier kann bei Bedarf einfach hochgeklappt werden (*DP 4*).

Eine Kalibrierung des Geräts auf den Nutzenden ist möglich, aber nicht zwingend erforderlich. Verbessert werden dadurch laut Microsoft u. a. der Tragekomfort sowie das Eye- und Handtracking. Zudem werden die ermittelten Werte nutzerspezifisch gespeichert, sodass die einmalige Kalibrierung des Geräts pro Nutzenden ausreicht. Die Steuerung erfolgt mithilfe von Hand- und Eyetracking oder Spracheingaben. Letztere sollen auch in lauten Umgebungen (z. B. Industrie) zuverlässig funktionieren (*DP 3*). Besonders wichtig für längere Tragezeiten ist die verbesserte Ergonomie der zweiten HoloLens-Generation. Das Gewicht zentriert sich nun nicht mehr an der Stirn, sondern wird auf Stirn und Hinterkopf verteilt (*DP 2*).

Die Batterielaufzeit hat sich gegenüber der ersten Generation nicht verbessert. Für den hier anvisierten Anwendungsfall der Hausbesuche ist eine Laufzeit von zwei bis drei Stunden jedoch ausreichend (*DP 1*).

Softwareseitig kommen Microsoft-eigene Lösungen zum Einsatz. Mithilfe von Dynamics 365 Guides können Anleitungen und Abläufe erstellt werden. Von Vorteil ist, dass dazu keine Programmierkenntnisse vonnöten sind. Die Anleitungen können im ersten Schritt per Software an einem herkömmlichen Computer erstellt und in einem zweiten Schritt mithilfe der Microsoft HoloLens 2 im Raum platziert werden. Während Routineuntersuchungen werden die Anleitungen situationsbezogen auf der MR Brille angezeigt und dienen so als Hilfestellung für das Fachpersonal. Wenn ein zahnmedizinisches Problem vorliegt, kann Dynamics 365 Guides genutzt werden, um die, für die Erfassung des Problems notwendigen Tätigkeiten Schritt für Schritt anzuzeigen.

Soll eine virtuelle Verbindung mit dem zahnärztlichen Personal erfolgen, wird diese über das Tool Remote Assist realisiert. Dies ermöglicht die Besprechung und Diskussion spontan auftretender Probleme sowie eine abschließende Kontrolle aus der Distanz heraus. Remote Assist bietet zudem innerhalb des Videotelefonats eine interaktive Toolbar. Dadurch kann die Person am Computer das Gespräch mit visuellen Hilfestellungen wie Freihandzeichnungen, Pfeilen oder Anmerkungen virtuell ergänzen oder gespeicherte Dokumente in einen gemeinsamen virtuellen Workspace laden.

Damit entspricht die hier beschriebene Umsetzung mit der Microsoft HoloLens 2 den Anforderungen an die spezifischen Einsatzszenarien sowie den, aus der Literatur abgeleiteten, Meta-Anforderungen und Designprinzipien.

## Diskussion und Fazit

Mithilfe von Gruppendiskussionen und einem Experteninterview konnten fünf praktische Einsatzszenarien für die Verwendung von MR Brillen im Rahmen zahnmedizinischer Hausbesuchen identifiziert werden. Darüber hinaus ergab eine systematische Literaturrecherche 14 genannte Probleme beim Einsatz von Mixed und Virtual Reality Brillen im medizinischen Bereich. Die von den Problemen abgeleiteten 14 Meta-Anforderungen wurden zu fünf Designprinzipien zusammengefasst. Diese Ergebnisse wurden abschließend in ein Umsetzungskonzept für die zuvor beschriebenen praktischen Einsatzszenarien überführt.

Im nächsten Schritt sollen die hier dargestellten Einsatzszenarien praktisch erprobt werden. Dafür wird zahnmedizinisches Fachpersonal für Hausbesuche mit der Microsoft HoloLens 2 und entsprechender Software ausgestattet. Das Augenmerk liegt dabei auf der Gebrauchstauglichkeit des Hardware‑/Softwaresystems sowie der Akzeptanz der Nutzenden und zu behandelnden Person. Die so gewonnenen Erkenntnisse helfen auch, die Designprinzipien zu evaluieren und bei Bedarf im Sinne des iterativen Vorgehens anzupassen.

Hier sind auch die Limitationen dieses Beitrags zu sehen. Die Designprinzipien fußen zwar auf der Literatur. Eine Evaluation steht allerdings noch aus. Ähnlich verhält es sich mit den beschriebenen Einsatzszenarien. Diese wurde mithilfe von verschiedenen Experten identifiziert und ausgearbeitet. Die technische Umsetzbarkeit ist gegeben. Ob sich das Konzept auch im Praxiseinsatz bewährt, hängt allerdings besonders auch von weiteren nicht-technischen Faktoren ab. MR Brillen gehören nicht zu alltäglichen Gegenständen, sodass bisher unsicher ist, wie die zu behandelnden Personen auf den Anblick reagieren. Hinzu kommen die Akzeptanz des Fachpersonals sowie weitere unbestimmte Variablen wie die Umgebung während des Hausbesuchs (Lichtverhältnisse, Infrastruktur, etc.). All diese Punkte bedürfen zu diesem Zeitpunkt noch der Evaluation.

Im Rahmen der Maßnahmen zur Einschränkung der Corona-Pandemie spielen Kontaktbeschränkungen eine entscheidende Rolle. Der Einsatz von Telemedizin ermöglicht die Aufrechterhaltung der medizinischen Versorgung bei gleichzeitiger Reduktion nicht notwendiger Kontakte. Das ärztliche Personal wird entlastet und die Patientensicherheit erhöht. Dies bietet im Endeffekt auch ein Kosteneinsparungspotential für die Krankenkassen.

Die in diesem Beitrag beschriebenen Probleme, Meta-Anforderungen und Designprinzipien zeigen technikaffinem Fachpersonal eine Umsetzungsmöglichkeit ihrer eigenen Projekte mit MR Brillen im medizinischen Bereich auf und definieren relevante Stolpersteine bei der Implementierung.

Der Hinweis auf diese aktuelle Forschungslücke stellt einen Erkenntnisgewinn zur Weiterentwicklung der wissenschaftlichen Thematik im Bereich Mixed und Virtual Reality Brillen dar. Zwar findet sich hier bereits Literatur zur Nutzung in den Disziplinen Chirurgie, Edukation und Angstpatienten. Die Verwendung während zahnmedizinischer Hausbesuche wurde allerdings noch nicht beforscht. Auch werden etwaige Anwendungsprobleme in der Literatur selten beschrieben (Sparwasser et al. 2018). Die hier dargestellten Erkenntnisse können daher als Ausgangsbasis für weitergehende Forschung genutzt werden.
